# Simulating Moral Actions: An Investigation of Personal Force in Virtual Moral Dilemmas

**DOI:** 10.1038/s41598-017-13909-9

**Published:** 2017-10-24

**Authors:** K. B. Francis, S. Terbeck, R. A. Briazu, A. Haines, M. Gummerum, G. Ganis, I. S. Howard

**Affiliations:** 1 University of Reading, Department of Philosophy, Whiteknights Campus, Reading, RG6 6AA United Kingdom; 2 University of Plymouth, School of Psychology, Drake Circus, Plymouth, PL4 8AA United Kingdom; 3 University of Plymouth, Transtechnology Research, Drake Circus, Plymouth, PL4 8AA United Kingdom; 4 University of Plymouth, Centre for Robotics and Neural Systems, Drake Circus, Plymouth, PL4 8AA United Kingdom

## Abstract

Advances in Virtual Reality (VR) technologies allow the investigation of simulated moral actions in visually immersive environments. Using a robotic manipulandum and an interactive sculpture, we now also incorporate realistic haptic feedback into virtual moral simulations. In two experiments, we found that participants responded with greater utilitarian actions in virtual and haptic environments when compared to traditional questionnaire assessments of moral judgments. In experiment one, when incorporating a robotic manipulandum, we found that the physical power of simulated utilitarian responses (calculated as the product of force and speed) was predicted by individual levels of psychopathy. In experiment two, which integrated an interactive and life-like sculpture of a human into a VR simulation, greater utilitarian actions continued to be observed. Together, these results support a disparity between simulated moral action and moral judgment. Overall this research combines state-of-the-art virtual reality, robotic movement simulations, and realistic human sculptures, to enhance moral paradigms that are often contextually impoverished. As such, this combination provides a better assessment of simulated moral action, and illustrates the embodied nature of morally-relevant actions.

## Introduction

*There is more reason in your body than in your best wisdom* ^1^. Moral dilemmas contrast the choice between individual rights and pursuing ‘the greater good’. Researchers from a variety of disciplines have long explored this issue in “thought experiments” that aim to understand and interpret moral decision-making. Perhaps the most acknowledged (or well-known) example of this is the “Trolley Problem”; incorporating two contrasting dilemmas, which have been vastly deliberated in the fields of philosophy, neuroscience, and psychology^2,3^. In the *switch* dilemma, individuals must decide whether to flick a switch to redirect a trolley car to kill one worker on the tracks rather than letting it proceed and kill five workers. Alternatively, in the *footbridge* dilemma, individuals must decide whether to push a man in front of the trolley, in order to stop it moving and thereby prevent it from killing the five workers. These two dilemmas are the subject of much debate, as individuals tend to endorse the utilitarian response (killing one to save many) in the “impersonal” *switch* case but refuse to do so in the “personal” *footbridge* case^4^.

Several theories have attempted to understand these divergent responses, given their structural similarity in entailing the five-for-one trade off^4^. One way to examine these distinct responses is to consider the moral principles that influence moral judgments^5^. For example, Greene, *et al*.^6^ found that if personal force–“the force that directly impacts the other [person] is generated by the agent’s muscles”^6^–was required in a dilemma, utilitarian judgments were significantly lower. This concept of personal force reaffirms the “simulated motor plan” hypothesis, suggesting that when faced with a personal dilemma, individuals imagine carrying out the harmful action^7^. In the *footbridge* dilemma for example, we might imagine ourselves pushing the person, shaping our hands and preparing our bodies in such a way as to direct our personal force onto them. In doing so, we are faced with an immediate emotional aversion to this act^2^ and so we judge it as unacceptable^7^. This theory relates back to the finding that responding in a utilitarian manner to personal dilemmas requires suppression of an affective response^2^. In a similar line of research, Cushman^8^ related personal force and physical contact back to reinforcement learning mechanisms. For humans, simply the act of pushing carries with it a history of learned moral violations. The *switch* dilemma on the other hand, does not contain personal force, involving the atypical moral action of flicking a switch. As such, it does not trigger the same aversion^2,8^.

In one experiment, Cushman, *et al*.^9^ utilised an active behavioural paradigm in order to investigate simulated harmful actions. In their study, the authors had participants simulate harmful actions such as hitting a plastic baby doll or hitting a PVC leg with a hammer, and found that individuals experience a strong aversion to performing these actions. They demonstrated that the degree to which individuals found simulating canonically harmful actions aversive, predicted the likelihood of endorsing a utilitarian moral judgment; the greater the degree of aversion in the behavioural paradigm, the less likely a subsequent utilitarian judgment^9^. However, one criticism of this behavioural paradigm is that harmful actions did not result in specific outcomes such as saving more lives^9^. As such, this paradigm may not offer a valid comparison to the classic one-for-many dilemmas frequently adopted in the moral domain^2^.

Recently Virtual Reality (VR) methods have been employed to study moral decision-making in which “the full repertoire of contextual features comes into play”^10^ allowing questioning of the theoretical and normative decisions in the framework of moral action^11–16^. In simulations of the *switch* dilemma, Patil, *et al*.^10^ found that utilitarian responses were greater in VR when action was required compared to text-based counterparts when judgments were required. This supports previous findings regarding the disparity between judgment and simulated action^17^. Furthermore, and in line with this, we recently incorporated the personal *footbridge* dilemma in a virtual paradigm^18^. Participants were either asked to simulate an action in this virtual dilemma (group one) or to respond to judgment questions in the theoretical version of the dilemma (group two). Overall, it was found that there were a greater proportion of utilitarian responses when action was required, as compared to the theoretical counterpart when judgments were required^18^. Anti-social personality traits, including psychopathy, predicted this endorsement of harm in VR, but did not predict utilitarian judgments in text-based counterparts, providing further evidence for the role of trait psychopathy in judgment-action discrepancy^19–21^. Despite this, research has yet to consider the impact of incorporating tools that allow the examination of physical contact and personal force in the context of simulated moral action. Whilst the virtual footbridge dilemma is “up close and personal”^2^, sensorimotor qualities and aspects of embodiment are still absent^15^. In order to address this limitation, in the following experiments, we incorporate haptic feedback within VR paradigms, examining its influence on simulated moral action.

## Experiment 1

Experiment 1 examined the relationship between moral judgment and simulated moral action, and more specifically, the influence of haptic feedback on simulated moral actions. Here, we use a robotic haptic interface (vBOT system) to simulate performing a realistic physical action in response to moral dilemmas involving personal force^22^. The vBOT system can simulate the physical resistance force that would be experienced from touching or pushing a physical object and is thus able to generate realistic haptic feedback. For example, it can generate the sensation of moving the hand through water or touching a spherical object. In the present experiment, we use the term ‘Haptic Virtual Reality’ or haptic VR to refer to any technology that can generate realistic sensations. As such, the vBOT is categorised here as a VR system since it can simulate making contact with virtual objects. Given that utilitarian endorsements have been found to be greater in moral action paradigms when compared to judgment counterparts^17^ and that virtual moral dilemmas often elicit a greater proportion of simulated utilitarian actions^10,14,18^, we predicted that utilising this virtual action framework would lead to an increase in simulated utilitarian responses. Previous research has argued that this response pattern arises from increased contextual saliency and a heightened focus on the negative consequences of inaction in VR^10,18,23^. Trait psychopathy may predict this increase, given findings that antisocial traits result in a greater proportion of utilitarian responses in action frameworks^19–21^.

## Methods

### Participants

Forty participants (34 females, 6 males) between 18 and 31 years (*M* = 20.23, *SD* = 2.97 years) were recruited from the Plymouth University (School of Psychology) participant pool and participated for course credit. All participants had normal or corrected-to-normal vision and were right-handed. This research received ethical approval from Plymouth University Ethics Committee and informed consent was obtained from all participants. All experiments were performed in accordance with the guidelines and regulations set out by the ethics committee.

### Measures

Participants were asked to fill out an electronic questionnaire comprising four self-report questionnaires:

The *Levenson Psychopathy Scale (LSRP)* ^24^ is a self-report measure of trait Psychopathy designed for non-institutionalised populations. It comprises a two-factor structure measuring both primary (i.e., callousness) (16 items; α = 0.90) and secondary psychopathic traits (i.e., impulsivity) (10 items; α = 0.66). The scale contains 26 items in total that are rated on a 4-point Likert scale (from 1 = *strongly disagree* to 4 = *strongly agree*). The scale includes items such as “*Success is based on survival of the fittest; I am not concerned about losers*”.

The *Hexaco-IP-PR* ^25^ is a personality inventory assessing six dimensions of personality. The inventory measures the following characteristics: Honesty-Humility (Items 10; α = 0.81), Emotionality (Items 10; α = 0.83), Extraversion (Items 10; α = 0.74), Agreeableness (Items 10; α = 0.87), Conscientiousness (Items 10; α = 0.80) and Openness to experience (Items 10; α = 0.78). The inventory contains 60 items, rated on a 5-point Likert scale (from 1 = *strongly disagree* to 5 = *strongly agree*). The inventory contains items such as “*I want people to know that I am an important person of high status*”.

### Moral Judgment and Simulated Action Measures

The between-groups experiment comprised two conditions to which participants were randomly allocated; a judgment condition (*N* = 20) and a simulated action condition (*N* = 20). In both conditions, participants were presented with vignettes describing the footbridge dilemma and 14 further personal force dilemmas. These moral dilemmas were selected from those originally used in Greene, *et al*.^2^ and also included five personal force dilemmas adapted from this database (see Supplementary Material: Appendix A online). Of the dilemmas originally used in Greene, *et al*.^2^, personal dilemmas were modified to ensure that each involved personal force. For example, consider the “euthanasia” dilemma:

*You are a leader of a small group of soldiers. You are on your way back from a completed mission deep in enemy territory when one of your men has stepped in a trap that has been set by the enemy and is badly injured. The trap is connected to a radio device that by now has alerted the enemy to your presence. They will soon be on their way*.

*If the enemy finds your injured man they will torture him and kill him. He begs you not to leave him behind, but if you try to take him with you your entire group will be captured. The only way to prevent this injured soldier from being tortured is to shoot him yourself*.

This dilemma, while categorised as personal, does not involve personal force according to its original definition^6^. As such, in the present experiment it was adapted as follows:

*If the enemy finds your injured man they will torture him and kill him. He begs you not to leave him behind, but if you try to take him with you your entire group will be captured. The only way to prevent this injured soldier from being tortured is to kill him yourself by stabbing him*.

Replacing a gun with a knife ensures that “the force that directly impacts the other [person] is generated by the agent’s muscles”^6^.

In both conditions, utilitarian and non-utilitarian responses were represented as a binary variable and the mean utilitarian proportion across all dilemmas was subsequently calculated for each individual by dividing the number of their utilitarian responses by the total number of dilemmas.

### Simulated Action Variables

In the simulated action condition, as well as generating an overall response to the dilemma, the vBOT system provided additional measures including force, speed and subsequent power.

#### Force

Baseline force measurements were calculated for each participant in order to control for varying strengths among participants. The vBOT arm allowed participants to push forward when simulating a utilitarian action or to pull away when refusing to endorse an action (non-utilitarian). A normalised force measure was subsequently calculated for the simulated utilitarian actions. Baselines measurements were first created by averaging the force of endorsements of actions in non-moral dilemmas (baseline force for endorsements). The normalized force applied by each participant when producing a simulated utilitarian response was then calculated as a proportion of their baseline force.

#### Speed

Speed was defined as the maximum speed (cm/s) that a participant moved the vBOT arm across the movement trajectory. Using the same procedure for force measurements, a normalised maximum speed was conditionally calculated for the simulated utilitarian actions. Baselines measurements were created by averaging the speed of endorsements of actions in non-moral dilemmas. The normalized speed was then calculated for the simulated utilitarian actions as a proportion of this baseline speed.

#### Power

The relative force and speed with which individuals simulated utilitarian actions were strongly correlated, (*r*(18) = 0.51, *p* = 0.021). Given that the product of speed and force equates to a measure of power, we used normalised force and speed scores to create a relative measure of power for each participant. This represented the power exerted by an individual when simulating a utilitarian action with the vBOT arm.

### Procedure

In both conditions, participants first completed the electronic questionnaire comprising the trait assessments. All personal force dilemmas were presented in a randomised order. In the judgment condition, dilemmas were presented to participants on a computer running E-Prime software and each dilemma was presented in three blocks of text that could be read at a speed determined by the participant. After each dilemma, participants were asked a morality question (“*Is it morally acceptable to* [*specific to the scenario*]?”) followed by a behavioural question (“*Would you do it?*”). Responses were given by selecting “Yes” (Y-key) or “No” (N-key). Participants were given 8 seconds to respond. This time frame was selected as it is long enough to allow responses that are not time-pressured but short enough to prevent a long elaborate decision-making process that would be unrealistic in an action framework. Similar time frame windows have been adopted in previous VR moral action paradigms^10,16^.

**Figure 1:**
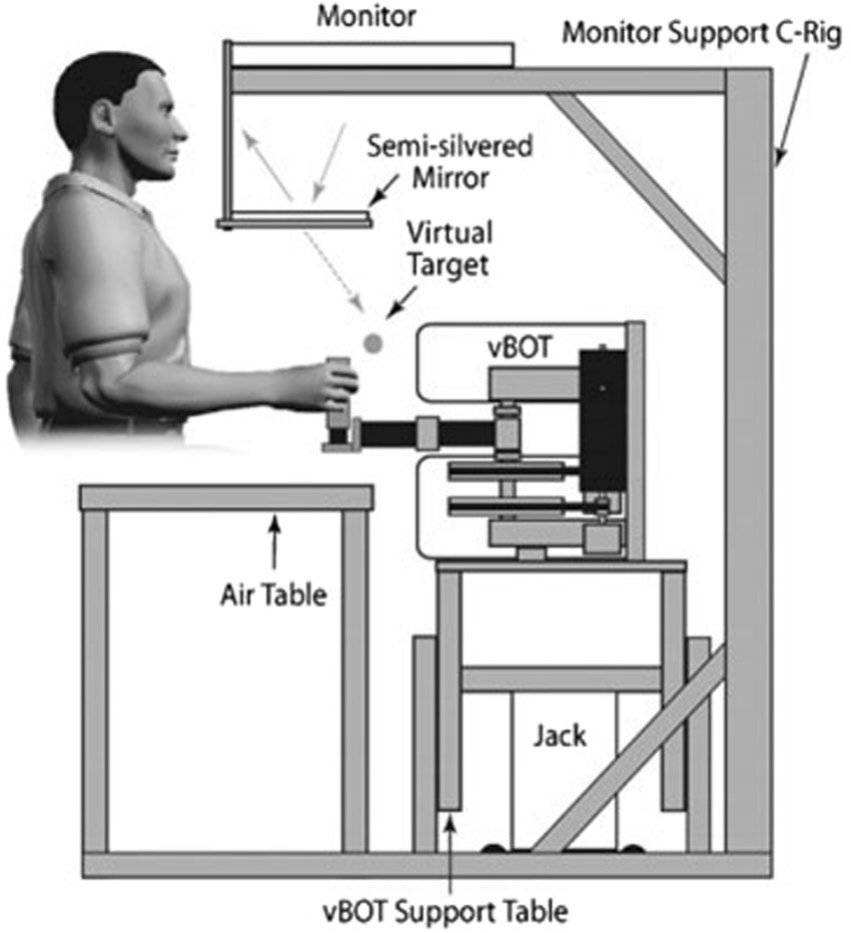
The vBOT System. The diagram shows the side-view of the set-up with a participant holding the handle of the vBOT arm with their right hand whilst viewing the monitor via a semi-silvered mirror. Text-based vignettes of dilemmas are displayed on the monitor for participants to read.

In the simulated action condition, participants initially completed an electronic pre-questionnaire assessing their gaming experience (hours per week of video game play and the number of games played annually). Participants were then presented with three non-moral dilemmas (see Supplementary Material: Appendix B online) selected from an existing database^2^ to provide a baseline measure of force. The personal-force moral dilemmas were then presented. All dilemmas were presented using the vBOT system (see Fig. 1).

At the start of each trial the vBOT handle first pulled the participant’s right hand to the starting position, which was at a central location (in the mid-sagittal plane 30 cm below the eyes and 30 cm in front of the chest). Participants were prevented from viewing their hand directly, and the VR system was used to overlay images of the hand cursor (0.5 cm radius red disk).

Participants were able to read the dilemmas in the semi-silvered mirror and as in the judgment condition, these were presented in blocks of text that could be read at a speed determined by the participant. Participants used a button press with their left hand to scroll forward through these blocks of text. After the end of each dilemma had been reached, upon a final left-hand button click the participant was asked (“*Are you going to* [*specific to scenario*]*?”*) followed by the phrase (*“If so*, *move the arm forward to* [*specific to scenario*]. *If not*, *then pull away* [*specific to scenario*]*”*). A final button press was then used to cue the response action and this generated the message (“*Act now*”). Matched to the judgment condition, after reading each dilemma in full, participants were given 8 seconds to respond.

A utilitarian endorsement was achieved when the handle was pushed forward into a soft object (which required the application of force) by more than 2 cm. The simulated soft object was located immediately forward of the start position and implemented using the combined effect of a weak spring (k = −4Ncm^−1^) and a resistive viscous field (k = −0.5Ncm^−2^). As such, when simulating a utilitarian response, the vBOT arm produced the physical resistance force that would be experienced when making contact with an object, thereby generating haptic feedback to the participant. A refused (non-utilitarian) response was achieved when the handle was pulled backwards more than 1.25 cm from the start position. No resistance was experience when pulling back. In this system, the handle position of the manipulandum is measured using optical encoders sampled at 1000 Hz and it uses motors operating under torque control to allow the application of end-point forces. A force transducer (Nano 25; ATI) is mounted under the handle to measure the applied forces.

In both conditions, if a response was omitted (no response was given after 8 seconds), the program would then move on to the next dilemma and the omitted scenario would be presented again at a later stage during the experiment. In the judgment condition, the proportion of utilitarian endorsements for morality and behavioural questions was recorded. In the simulated action condition, the proportion of utilitarian actions, force, and speed were all recorded for further analyses. Response times in the judgment and simulated action conditions were recorded by different systems and as such, could not be directly compared.

### Data availability

The datasets generated during and/or analysed during the current investigation are available from the corresponding author on reasonable request.

## Results

### Pre-questionnaire Responses

In the simulated action condition, it was found that generating a utilitarian moral action using the vBOT system was not associated with previous gaming experience, as shown using bivariate Spearman correlations comparing utilitarian actions and hours per week of video game play, (*r*_*s*_(18) = 0.25, *p* = 0.289) and number of games played annually, (*r*_*s*_(18) = 0.38, *p* = 0.096).

### Moral Responses

**Figure 2:**
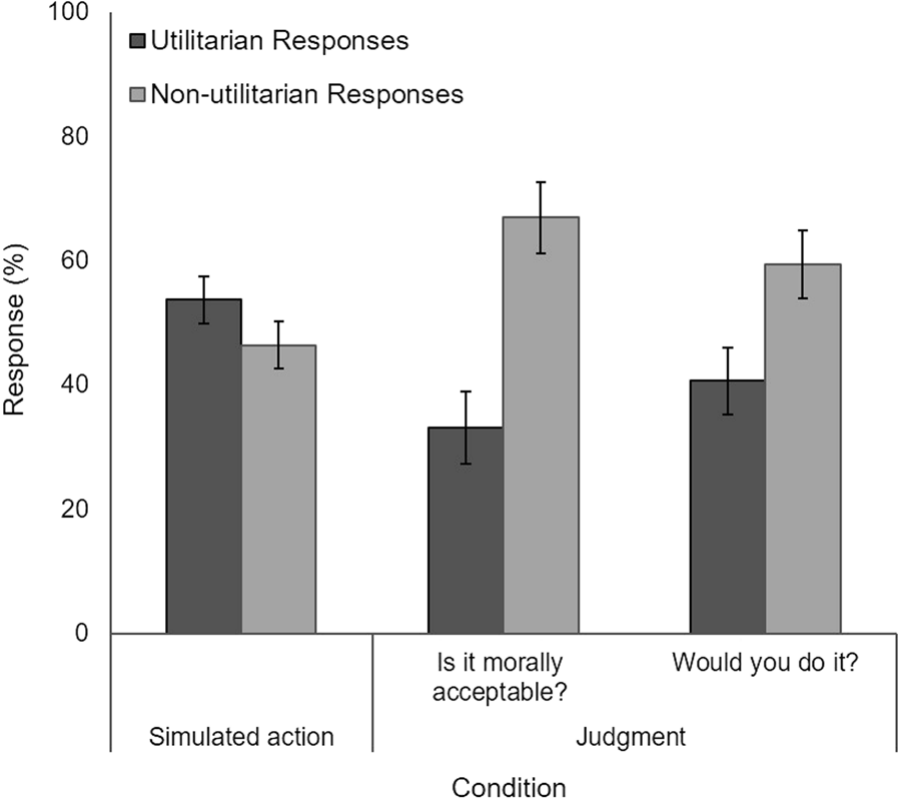
Simulated Moral Action and Moral Judgment Responses. Responses (%) (utilitarian or non-utilitarian) in the simulated action condition and judgment condition in response to personal force moral dilemmas. In the judgment condition, participants were asked both a morality question and behavioural question. A greater number of utilitarian endorsements were observed in the simulated action condition when the vBOT system was used to generate actions. Error bars represent +−1 SE.

Analyses compared responses to personal-force moral dilemmas in the simulated action condition (simulated utilitarian actions using the vBOT system) versus the judgment condition. In the simulated action condition, participants endorsed a greater proportion of utilitarian actions in personal force dilemmas (*M* = 0.54, *SD* = 0.17) compared to the judgment condition when responding to both the morality question (*M* = 0.33, *SD* = 0.26) and the behavioural question (*M* = 0.41, *SD* = 0.24) (see Fig. 2).

#### Morality question: Is it morally acceptable?

We conducted a one-way ANOVA with condition (simulated action; judgment) as the between-subjects factor. In this ANOVA, the morality question was utilised as the dependent variable for the judgment condition.

The assumption of homogeneity of variance was violated and as such, the Brown-Forsythe *F*-ratio is reported. Analysis revealed a main effect of condition, (*F*(1, 32.64) = 8.89, *p* = 0.005, *η*_*p*_ *²* = 0.20) with a greater proportion of utilitarian endorsements observed in the simulated action condition compared to the judgment condition.

#### Behavioural question: Would you do it?

We conducted a one-way ANOVA with condition (simulated action; judgment) as the between-subjects factor. In this ANOVA, the behavioural question was utilised as the dependent variable for the judgment condition.

Analysis revealed a marginally significant main effect of condition, (*F*(1, 38) = 3.92, *p* = 0.055, *η*_*p*_ *²* = 0.10) with a greater proportion of utilitarian responses observed in the simulated action condition compared to the judgment condition.

In the judgment condition specifically, no significant difference was found when comparing responses to the morality question (i.e., moral acceptability) and the behavioural question (i.e., whether they would do it), (*p* = 0.378).

### Personality Trait Analyses

In order to assess any differences between the personality traits of participants in both the judgment and simulated action conditions, a one-way ANOVA was used to compare trait measures. No significant differences between the judgment and simulated action conditions were found (all *ps* > 0.071), except for Honesty-Humility, (*F*(1, 38) = 6.31, *p* = 0.016, *η*_*p*_ *²* = 0.14) which was higher in the judgment condition (*M* = 3.63, *SD* = 0.47) than the simulated action condition (*M* = 3.17, *SD* = 0.67).

#### Traits and action variables

**Figure 3:**
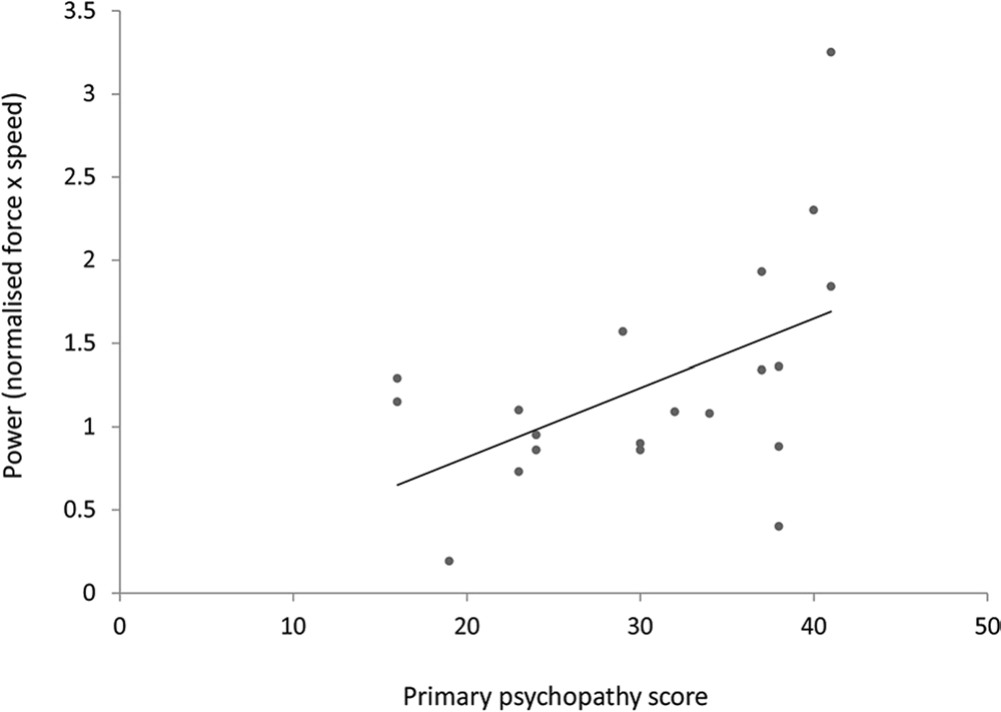
Primary Psychopathy Scores and Power. Primary psychopathy scores plotted against power exerted when simulating utilitarian (harmful) moral actions with the vBOT. The power exerted when simulating a utilitarian (harmful) action with the vBOT, was positively correlated with primary Psychopathy score. *Note*. The high reading at 3.25 power was investigated following visual inspection. Given that Spearman’s rho is robust to these univariate outliers^30^, correlational analyses were repeated using this procedure. Power continued to be positively associated with primary psychopathy, (*r*_*s*_(18) = 0.45, *p* = 0.045).

**Figure 4:**
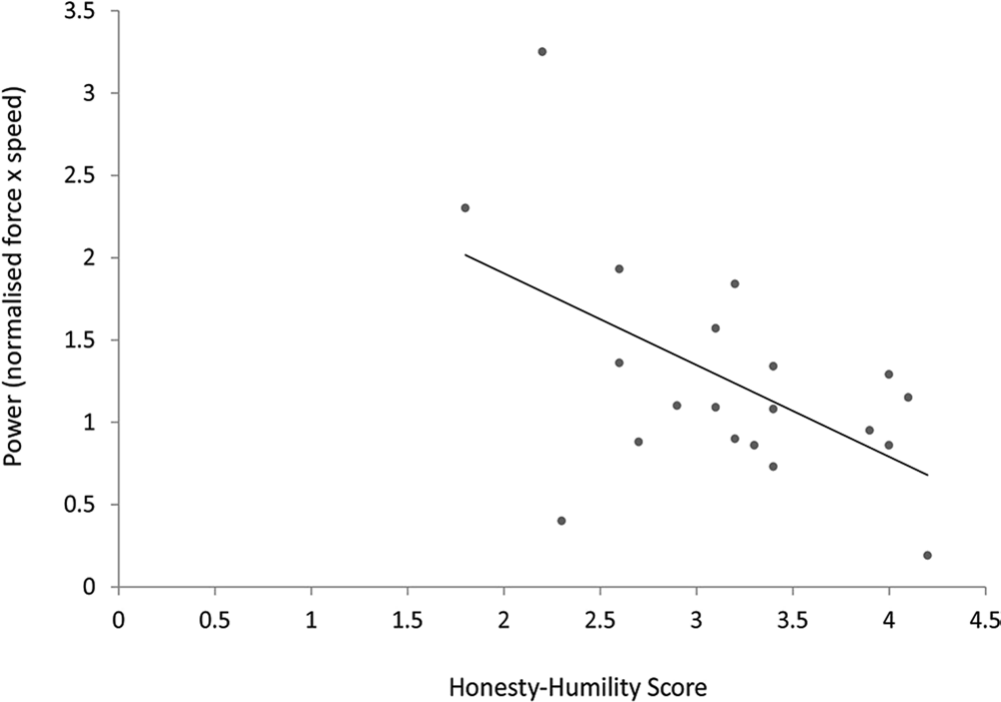
Honesty-Humility Scores and Power. Honesty-Humility scores plotted against power exerted when simulating utilitarian (harmful) moral actions with the vBOT. The power exerted when simulating a utilitarian (harmful) action with the vBOT, was negatively correlated with Honesty-Humility score. *Note*: The robustness of this relationship was examined in light of the high reading at 3.25 power. As such, correlational analyses were replicated using Spearman’s rho. Power continued to be negatively associated with Honesty-Humility, (*r*_*s*_(18) = −0.47, *p* = 0.039).

Bivariate correlations revealed that traits did not relate to utilitarian responses in either the judgment or the simulated action conditions directly (*ps* > 0.109). However, in the simulated action condition specifically, in order to determine whether personality traits were related to the physical power (force × speed) of utilitarian actions, we conducted bivariate correlations between traits and the power exerted when performing a utilitarian action (simulating a harmful act). Correlations revealed a significant positive correlation between power exerted and primary Psychopathy, (*r*(18) = 0.51, *p* = 0.022) and a significant negative correlation between power exerted and Honesty-Humility, (*r*(18) = −0.55, *p* = 0.013). In order to determine whether these traits predicted the power exerted by each participant, two univariate regressions were conducted. When the LSRP dimensions were entered as continuous predictors and proportion of force endorsed as the outcome variable, primary Psychopathy (LSRP dimension) was found to explain 26% of the variance in the model, (*R* ^2^ = 0.260, *F*(1,18) = 6.33, *p* = 0.022) predicting the power exerted when simulating utilitarian actions using the vBOT system (*β* = 0.04, *p* = 0.022) (see Fig. 3). Honesty-Humility, was a significant negative predictor of power exerted (*β* = −0.56, *p* = 0.013) when entered in an additional univariate regression with all HEXACO traits, explaining 30% of the variance in the model, (*R* ^2^ = 0.298, *F*(1,18) = 7.65, *p* = 0.013) (see Fig. 4).

## Experiment 2

In Experiment 1, whilst we included a haptic device (vBOT system) in order to allow simulations of moral actions, we are aware of the limitations of relying on text-based formulations of personal-force dilemmas. Previous research examining simulated murder has predominantly incorporated life-like stimuli such as PVC arms^9^, whereas Experiment 1 relied on text and the use of a more abstract response device (vBOT arm) which may “…lack salient properties reliably associated with victim distress”^9^. As such and in order to examine the combined effects of visual immersion in state-of-the-art VR technologies and haptic feedback based on realistically simulated moral actions, Experiment 2 combined the visually immersive virtual version of the *footbridge* dilemma described in previous research^4,18^ and an interactive sculpture mechanism designed to generate haptic feedback and the sensation of pushing the person off the footbridge.

## Methods

### Participants

Twenty-five participants (13 females, 12 males, *M*_*age*_ = 33.80, *SD* = 13.51 years, age range: 19–64 years) were recruited from the public in Plymouth and the surrounding area of Devon and took part on a voluntary basis during a public engagement event at Plymouth University. Data were collected in a separate room divided from the main foyer of the event, with the primary investigator and a research collaborator present. This research received ethical approval from Plymouth University Ethics Committee and informed consent was obtained from all participants. All experiments were performed in accordance with the guidelines and regulations set out by the ethics committee.

### Moral Action Measure

**Figure 5:**
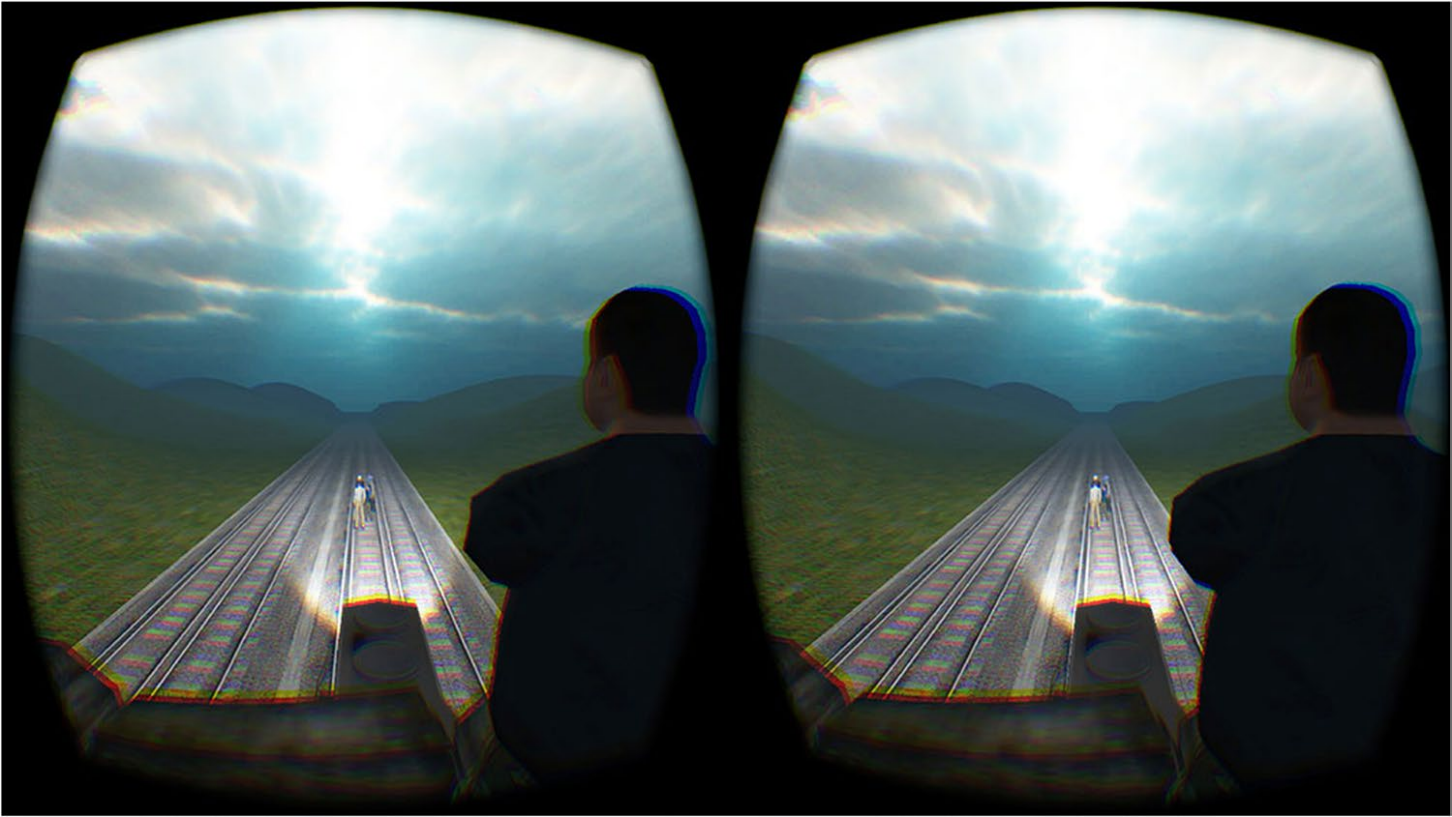
The Virtual Footbridge Dilemma. Stereoscopic image showing a scene from the footbridge virtual dilemma through Oculus Rift head-mounted display as seen in Francis *et al*.^16^. The image is taken from the viewpoint of the participant at the end of the scenario in which the trolley car is about to collide with the virtual avatars standing on the tracks ahead. Participants are able to rotate in the virtual environment and voice commands are included to ensure full understanding of the events playing out.

**Figure 6:**
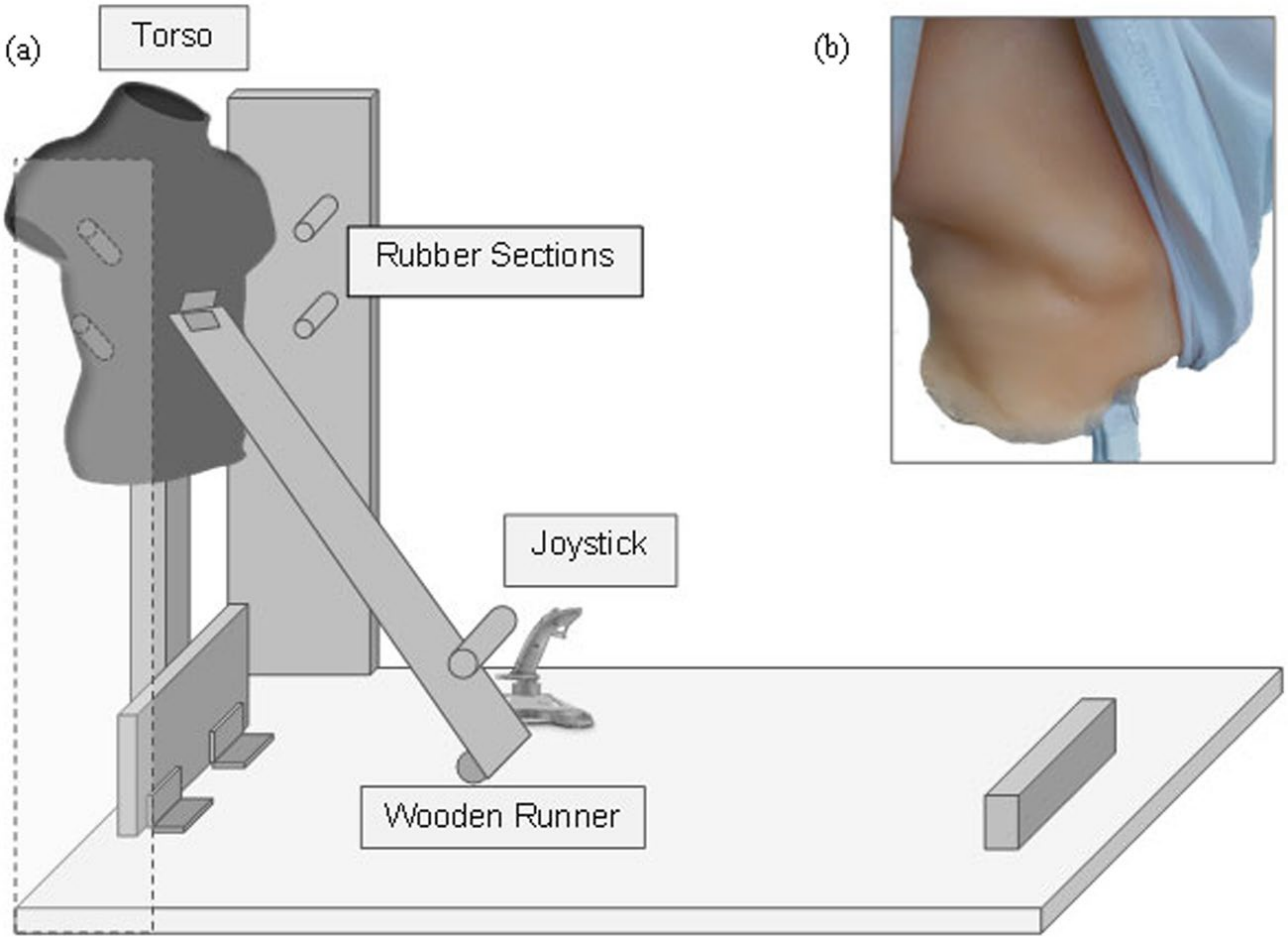
The Interactive Sculpture Mechanism. (**a**) The diagram shows the side-view of the interactive sculpture. The participant would stand behind the back of the torso wearing the Oculus Rift. If they chose to endorse a utilitarian action, they would then apply pressure to the back of the torso with their own hand. Rubber sections on the outside of the sculpture would generate resistance when pushed and would spring forward to release the body of the sculpture if pushed hard enough. A wooden runner would then capture the weight of the body as it fell, pushing the joystick forward, subsequently triggering the virtual action of pushing in the VR headset. (**b**) Photograph displaying the silicon beneath the fabric covering the back of the torso. Heated wiring sits beneath this silicon layer generating a body-like temperature.

The virtual version of the *footbridge* dilemma^18^ is an audio-visual virtual scenario presented to participants via a head-mounted display. The *footbridge* dilemma as described in Foot^3^ plays out in real-time with the participant standing on a footbridge behind a large virtual human (see Fig. 5). Verbal instructions are played during the scenario describing the situation as it plays out, finishing with the statement (“*If you’re going to push them*, *do it now*, *but it is your choice*”). In the original experiment, as described in Francis, *et al*.^18^, participants were then given a maximum of ten seconds to respond in the dilemma by either pushing the person with a joystick device or by choosing to do nothing. In the present experiment, all elements of the virtual dilemma were kept the same including the audio descriptions and response time. However, the joystick device was replaced with an interactive sculpture mechanism. As part of a multidisciplinary project, the interactive sculpture was designed in the shape of a large person’s back (see Fig. 6). This response device had several key features designed to generate haptic feedback and create an immersive experience for participants:

(i). The body of the sculpture itself was created using expandable foam and finished with platinum grade silicon. When fabric was placed over this textured surface, the feeling of the sculpture mirrored that of a real person. Heated wiring was also built in beneath the silicon coating to warm the sculpture, again generating a life-like touch (see Fig. 6).
(ii). Sections of rubber were added to the front of the sculpture ensuring that enough resistance would be generated if someone attempted to push the sculpture forward. These rubber sections would hit the surrounding frame of the sculpture if it was only pushed tentatively. If pushed hard enough and with a more realistic force, the rubber sections would move past the frame, causing the sculpture to fall.
(iii). Upon falling, the sculpture would trigger the joystick mechanism, resulting in the person in the virtual dilemma falling off the bridge. This established synchronisation between the physical sensation of pushing and seeing the person fall in VR.

## Procedure

Participants were given the pre-questionnaire incorporated within Experiment 1 assessing previous gaming experience. Participants then entered a quiet room and were asked to place the Oculus Rift headset on and a pair of Sennheiser headphones. Subsequently, participants were guided forward into a separate room which held the interactive sculpture. This set-up was designed to ensure that participants did not see the sculpture in real-life, ensuring an immersive experience. Based on the participants’ height, they were placed at the correct distance from the sculpture in order to synchronise the sculpture location in real-life with the virtual person’s location in VR. Once the virtual dilemma had loaded, participants were verbally informed of the following; “*You can interact with the person standing in front of you*, *by reaching out with your right arm*”. Upon hearing this information, participants would extend their right arm and make contact with the interactive sculpture familiarizing them with its location. No further information was given following this and participants continued to listen to the audio descriptions as the scenario played out in VR as described in Francis, *et al*.^18^.

### Comparative Conditions

We compared responses made using this setup (haptic-VR action condition) to responses collected in Experiment 2 of the original paper incorporating the virtual *footbridge* dilemma (VR action condition) and text-based *footbridge* dilemma (judgment condition)^18^. This population was deemed an adequate comparative sample having also been sampled from the public in Plymouth and the surrounding area, with similar gender and age ratios^18^. In the VR action condition, participants completed the VR *footbridge* dilemma, as described above, but using a joystick device. A utilitarian endorsement was given by pushing the joystick forward and a non-utilitarian decision was given by choosing to do nothing. In the judgment condition, participants responded to the text-based *footbridge* dilemma and as in Experiment 1, responded to both a morality question (“*Is it morally acceptable to* [*specific to the scenario*]?”) followed by a behavioural question (“*Would you do it?*”). Responses were given by selecting “Yes” (Y-key) or “No” (N-key).

## Results

### Pre-questionnaire Responses

It was found that simulating a utilitarian action in the haptic-VR action condition using the interactive sculpture was not associated with previous gaming experience, as shown using bivariate Spearman correlations comparing utilitarian actions and hours per week of video game play, (*r*_*s*_(23) = 0.29, *p* = 0.163) and number of games played annually (*r*_*s*_(23) = 0.28, *p* = 0.177).

### Moral Responses

We compared responses from the haptic-VR action condition (*N* = 25) from the present research to responses from the VR action condition (*N* = 30) and judgment condition (*N* = 30) from previous research^18^. In the judgment condition, when asked if the act was morally acceptable, 10% of participants endorsed a utilitarian response (i.e. judge that they regard pushing the person as morally acceptable). In the VR action condition, 63.3% of participants generated a simulated utilitarian response (i.e. pushed the person off the bridge using the joystick). In the haptic-VR action condition in the present experiment, 56% of participants generated a simulated utilitarian response (i.e. pushed the person off the bridge using the interactive sculpture). A chi-square analysis revealed a significant difference between these three conditions, (χ²(2) = 20.18, *p* < 0.001).

**Figure 7:**
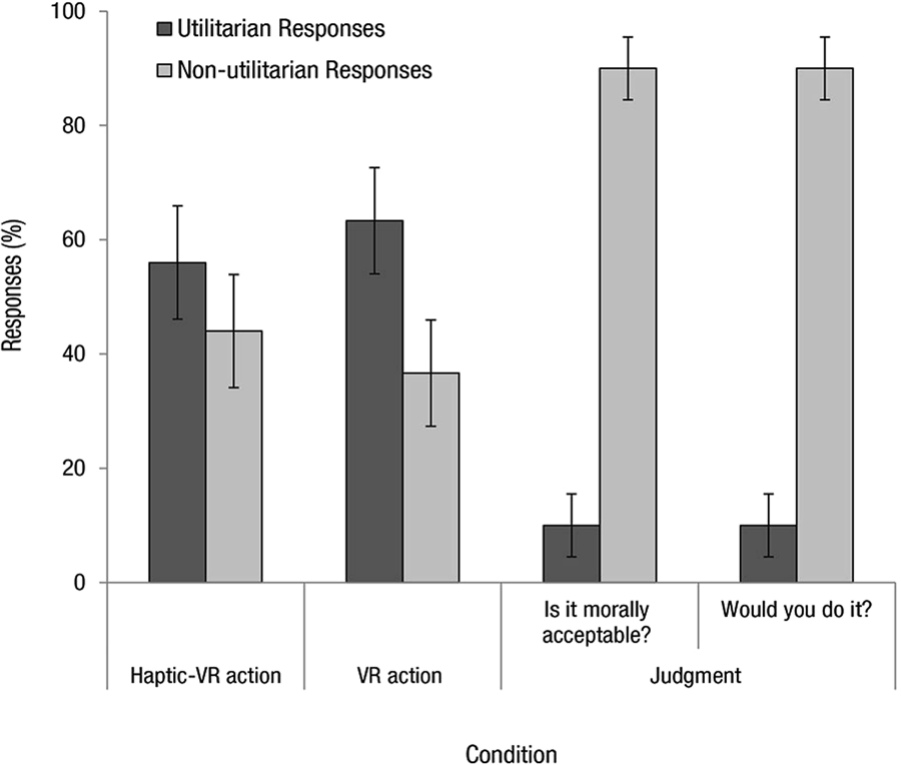
Moral Action and Moral Judgment Responses. Responses (%) in the haptic-VR action condition from Experiment 2 and VR action and judgment responses as reported in Francis, *et al*.^14^. In the judgment condition, participants were asked whether the action was morally acceptable and whether they would do it. In the VR action condition, participants completed the VR *footbridge* dilemma and responded using a joystick. In the Haptic-VR action condition, participants responded to the VR *footbridge* dilemma using the interactive sculpture mechanism. A greater number of utilitarian actions were observed in both the haptic-VR action and VR action conditions. Error bars represent +−1 SE_p_.

We used chi-square follow-up tests with Bonferroni corrections (*p* < 0.016) to determine which conditions were significantly different. As reported in Francis, *et al*.^18^, utilitarian endorsements were significantly higher in the VR action condition compared to the judgment condition, (χ²(1) = 18.37, *p* < 0.001). Utilitarian endorsements were also significantly higher in the new haptic-VR action condition when compared to the judgment condition, (χ²(1) = 13.51, *p* < 0.001). The odds of participants producing a simulated utilitarian response were 11.55 times higher in the VR-haptic condition than in the judgment condition. There was no significant difference between moral actions in the VR action condition from Francis, *et al*.^18^ and the moral actions in the new haptic-VR action condition (*p* > 0.580). When asked if they would perform the action (behavioural question) in the judgment condition, the same responses were observed with 10% of participants endorsing a utilitarian response in the text-based *footbridge* dilemma, compared to the 63.3% who endorsed the action in the VR action condition, (χ²(1) = 18.37, *p* < 0.001) and the 56% of participants who endorsed the action in the new haptic-VR action condition, (χ²(1) = 13.51, *p* < 0.001) (see Fig. 7).

## General Discussion

In two studies, we observed greater utilitarian responses to moral dilemmas when they were presented as physically and visually salient simulations. In Experiment 1, individuals receiving haptic feedback by simulating actions using the vBOT system, demonstrated a higher endorsement of utilitarian action when compared to individuals who made moral judgments within the traditional paradigm. In Experiment 2, when visually immersive VR was combined with a life-like response device that felt like a human body, greater simulated utilitarian action was again observed in response to the moral dilemma. While the pattern of moral judgments collected in the present investigation supports existing models of moral judgment, demonstrating fewer utilitarian responses to personal moral dilemmas^2,6^, the pattern of simulated moral action appears to sit in contrast, with greater utilitarian actions observed in personal dilemmas. In previous VR paradigms, this preference for generating simulated utilitarian action in VR has been linked to contextual saliency; the physical features of VR allow agents to “see” victims and this alters the way in which actions and outcomes are evaluated in moral dilemmas^10,18^. Greater negative emphasis may be placed on witnessing victims die rather than on producing simulated harmful actions, subsequently resulting in greater utilitarian responses^8,10,18^. Whilst this theory may explain the findings from Experiment 2, it fails to fully explain the results of Experiment 1 when visual components of VR were absent. When utilising the vBOT system, participants were not able to “see” victims, yet greater preference for making a simulated utilitarian action remained. As such, an additional explanation of the present findings is required. One possible explanation for this could rest in frame of reference accounts of judgment versus actions^17^. According to these accounts, action choices are driven by egocentric perspectives and unlike judgments, may overlap with self-interested motivations as individuals consider the self-relevant consequences of their actions^17,19^. Judging, on the other hand, relies on allocentric considerations influenced by cultural norms^17^. As such, the present research suggests that the judgment-action discrepancy observed here, appears to result from a combination of contextual salience^10,18^ and frame of reference accounts^17^.

While previous research has found a relationship between anti-social and pro-social personality traits and simulated moral action^10^, in Experiment 1, psychopathy was found to predict the power exerted when simulating a harmful action rather than the harmful actions themselves. Honesty-Humility, a pro-social trait negatively associated with the Dark Triad (a set of traits associated with malevolent qualities)^26^, negatively predicted this power. Research has increasingly pointed to the ambiguity of the motives underlying the responses given in trolley-type dilemmas; given that they may not derive from a moral concern but may stem from a lack of aversion to harm in certain individuals^27,28^. Perhaps, the power with which a moral action is simulated may provide a sensitive measure of the non-moral motivations that drive so-called “moral” actions in these virtual frameworks, beyond that of behavioural moral responses. However, these conclusions are given tentatively and future research should consider adopting approaches from differential psychology in order to explore the role of pro- and anti-social traits further with larger sample sizes.

Given the combination of the methodological approaches introduced here, we also explore additional interpretations of the present findings that need addressing. Firstly, the suggestion that the prevalence of simulated utilitarian actions observed may derive from gaming-related behaviours is not supported in the current research. Simulated moral actions were not predicted by self-assessments of game-related experiences (hours per week of video game play and number of games played annually) in either Experiment 1 or 2 suggesting that the responses simulated with the vBOT and interactive sculpture were not akin to desensitized actions often found in gaming contexts^18^. Additionally, previous research assessing gaming affordance effects found that the incorporation of joystick devices did not influence simulated moral actions^18^ and therefore it is unlikely that the vBOT arm itself in Experiment 1 or the interactive and life-like sculpture in Experiment 2, influenced subsequent simulated moral actions. Secondly, it is also important to note that differences existed between the judgment and simulated action conditions in Experiment 1, in terms of the framing of the judgment-based or action-based instructions. Specifically, in the simulated action condition, when responding to dilemmas using the vBOT system, participants were presented with the phrase *“Are you going to* [*specific to scenario*]*?”* prior to responding whereas participants in the judgment condition were presented with a morality and behavioural question. However, results from a follow-up experiment (Supplementary Material: Appendix C online) failed to support the presence of potential framing effects. Therefore, it is unlikely that the prevalence of utilitarian actions simulated with the vBOT system were generated by coercive framing effects. Further, in Experiment 2 we demonstrate that preference for utilitarian actions remains when any possible text-based framing effects are removed altogether and replaced with visually immersive VR. Finally, it is also important to consider whether individuals are just more likely to act in immersive or haptic VR than in a text-based or theoretical situation. However, this explanation is not likely, given recent evidence that people are more likely to risk their own lives to save someone else in text-based vignettes, than in a VR counterpart condition^29^. If individuals were simply more likely to act in VR, then we would expect to see the opposite pattern, with more people acting altruistically in VR^29^.

It terms of addressing limitations, it is important to note that in Experiment 1, Honesty-Humility scores were higher in the judgment condition, than in the simulated action condition and that arguably, this could be responsible for the differences in moral judgments and simulated moral actions. However, we would argue that these group differences in trait score were not likely responsible for the judgment-action discrepancy observed here, based on similar findings from previous virtual research also supporting this judgment-action discrepancy^10,14,18^. Further, reaction times were not compared across simulated action and judgment conditions as a result of differences in recording sensitivities. Given that reaction times can shed light on the mechanisms motivating convergent moral responses^2^, future research investigating judgment-action discrepancy should compare reaction times for judgment versus simulated action paradigms as an additional measurement of interest.

In Experiment 2, it is important to raise concerns regarding the sense of presence generated by the combination of the interactive sculpture and the immersive virtual environment. In the present paradigm, no form of body tracking was included to ensure that the sculpture was precisely aligned with the virtual avatar in the scenario (alignment was completed manually based on each participant’s height and position). Furthermore, in the virtual environment, participants could not see their own body in virtual form. These limitations may have compromised the level of immersion that could be generated in the experiment and as such, future research should incorporate accurate tracking between real objects and their virtual counterparts to control for this potential confound. In addition, given that moral actions simulated using the interactive sculpture and VR paradigm in Experiment 2 were collected during a public engagement event, we were unable to assess personality traits. As such, future research could investigate simulated moral actions using these multidisciplinary approaches in lab-based settings in which personality traits can also be assessed. In terms of sampling, whilst we acknowledge the limitation of comparing data collected at a public engagement event to data collected in the lab at a different time, this was not likely a confound; sample characteristics were similar having been collected from the public in Plymouth and the surrounding area.

Despite these shortcomings, the approaches to the investigation of simulated moral actions presented in this research, could offer insights into the embodied nature of morally-relevant actions beyond that of traditional moral judgment paradigms. It is important to note that in the present investigations, we did not aim to predict real-life behaviours from these experiments but rather to investigate the disparity between simulated moral action and moral judgment in controlled investigations of morality of harm. As such, we recommend that future research consider how these implicit measures of simulated moral action might predict real-world moral behaviours, expanding this research beyond hypothetical instances of simulated moral action. Although previously alien to the field of moral cognition and at the outset, these simulated moral action frameworks have allowed us to investigate the embodied nature of personal force and physical contact with greater significance than previously possible. Importantly, additional measures such as physical power, have offered us sensitive insights into high emotionally arousing moral scenarios, beyond those obtained in judgment-based paradigms. For example, researchers can now begin to investigate not only whether a simulated moral action was endorsed but how hard that action was simulated and what this might reflect about a person’s personality profile.

## Electronic supplementary material

Supplementary Material
