# Supplementary material for: Simulating Moral Actions: An Investigation of Personal Force in Virtual Moral Dilemmas
Source: Sci Rep. 2017 Oct 24;7:13954. doi: 10.1038/s41598-017-13909-9 (PMC5654774; doi:10.1038/s41598-017-13909-9)
Supplement: Supplementary file 1 — Supplementary Material [file 41598_2017_13909_MOESM1_ESM.pdf]

Simulating Moral Actions: An Investigation of Personal Force in Virtual Moral Dilemmas

Francis, K. B[1,*]., Terbeck, S[1]., Briazu, R. A[1]., Haines, A[2]., Gummerum, M[1]., Ganis, G[1]., & Howard, I. S[3]

[1]University of Plymouth, School of Psychology, Drake Circus, Plymouth, PL4 8AA, United Kingdom
[2]University of Plymouth, Transtechnology Research, Drake Circus, Plymouth, PL4 8AA, United Kingdom
[3]University of Plymouth, Centre for Robotics and Neural Systems, Drake Circus, Plymouth, PL4 8AA, United Kingdom
*corresponding author: kathryn.francis@plymouth.ac.uk

Supplementary Material

APPENDIX A: Personal Force Moral Dilemmas

Bomb

You are negotiating with a powerful and determined terrorist who is about to set off a bomb in a crowded area. Your one advantage is that you have his teenage son in your custody.

There is only one thing that you can do to stop him from detonating his bomb, which will kill thousands of people if detonated. To stop him, you must contact him over satellite hook-up that he has established and, in front of the camera, punch his son in the face, breaking his nose and then threaten to do worse if he does not give himself up.

Crocodiles

You are on a safari cruise that seems to be attracting the attention of several crocodiles, which begin to attack the boat. The force of the crocodiles and their attempts to board the boat, are causing it to sink. The boat is now sitting low in the water – a few inches lower and the crocodiles will be able to reach everyone on board.

The crocodiles become more aggressive. If nothing is done the boat will sink and the crocodiles will attack everyone on board, killing them. However, if you push one person overboard, the crocodiles will attack them leaving the boat to stay afloat. The remaining passengers will be saved.

Crying Baby

Enemy soldiers have taken over your village. They have orders to kill all remaining civilians. You and some of your townspeople have sought refuge in the cellar of a large house. Outside you hear the voices of soldiers who have come to search the house for valuables.

Your baby begins to cry loudly. You press your hand over his mouth to block the sound. If you stop pressing, his crying will summon the attention of the soldiers who will kill you, your child, and the others hiding out in the cellar. To save yourself and the others you must smother your child to death.

Euthanasia

You are a leader of a small group of soldiers. You are on your way back from a completed mission deep in enemy territory when one of your men has stepped in a trap that has been set by the enemy and is badly injured. The trap is connected to a radio device that by now has alerted the enemy to your presence. They will soon be on their way.

If the enemy finds your injured man they will torture him and kill him. He begs you not to leave him behind, but if you try to take him with you your entire group will be captured. The only way to prevent this injured soldier from being tortured is to kill him yourself by stabbing him yourself.

Fire

You are in a house with several people. A fire has started and you and the other people are trapped in a back room. You have called the emergency services but you know that it will take several minutes for you to be rescued. The fire is spreading quickly through the house and towards the room you have taken refuge in.

The fire is spreading dangerously quickly now and it is going to reach the room before you and the people can be rescued. The only way to save the people is to push one person into the fire, slowing its progress and leaving time for the emergency services to get to the house.

Footbridge

A runaway trolley is heading down the tracks toward five workmen who will be killed if the trolley proceeds on its present course. You are on a footbridge over the tracks, in between the approaching trolley and the five workmen. Next to you on this footbridge is a stranger who happens to be very large.

The only way to save the lives of the five workmen is to push this stranger off the bridge and onto the tracks below where his large body will stop the trolley. The stranger will die if you do this, but the five workmen will be saved.

Lawrence of Arabia

You are the leader of a small army that consists of warriors from two tribes, the hill tribe and the river tribe. You belong to neither tribe. During the night a hill tribesman got into an argument with a river tribesman and murdered him. The river tribe will attack the hill tribe unless the murderer is put to death, but the hill tribe refuses to kill one of its own warriors.

The only way for you to avoid a war between the two tribes that will cost hundreds of lives is to publicly execute the murderer by cutting off his head with your sword.

Lifeboat

You are on a cruise ship when there is a fire on board, and the ship has to be abandoned. The lifeboats are carrying many more people than they were designed to carry. The lifeboat you're in is sitting dangerously low in the water – a few inches lower and it will sink.

The seas start to get rough, and the boat begins to fill with water. If nothing is done it will sink before the rescue boats arrive and everyone on board will die. However, there is an injured person who will not survive in any case. If you push that person overboard the boat will stay afloat and the remaining passengers will be saved.

Mercy

You are the law enforcer of a small tribe. A prisoner has escaped from a nearby tribe's camp and he has fled into your community. After arresting him and interviewing him, you establish that his imprisonment was unjust. You know that the prisoner's escape has led to unrest in the other tribe and a mob is on its way.

If the mob finds the prisoner, they will torture him and kill him. He begs you not to hand him over to them, but if you try to protect him your entire tribe will be attacked by the approaching mob. The only way to prevent this prisoner from being tortured and to protect your tribe is to kill him yourself with your knife.

Mining

You are part of a mining expedition. A cave-in has left you and the other miners trapped. You have established radio contact with the surface but you know that it will take several hours for you to be rescued. The cave-in has limited your oxygen supply and has injured one of the miners who is quickly losing blood. The injured crew member is going to die from wounds no matter what happens.

The remaining oxygen is not sufficient for the all of the miners to make it to the surface. The only way to save the miners is to kill the injured miner so that there will be just enough oxygen for the rest of the miners to survive until you are rescued. There is a large rock beside you.

Sacrifice

You, your husband, and your four children are crossing a mountain range on your return journey to your homeland. You have inadvertently set up camp on a local clan’s sacred burial ground.

The leader of the clan says that according to the local laws, you and your family must be put to death. However, he will let yourself, your husband, and your three other children live if you yourself will kill your oldest son by pushing him off a nearby cliff face.

Safari

You are part of a group of ecologists who live in a remote stretch of jungle. The entire group, which includes eight children, has been taken hostage by a group of paramilitary terrorists. One of the terrorists takes a liking to you. He informs you that his leader intends to kill you and the rest of the hostages the following morning.

He is willing to help you and the children escape, but as an act of good faith he wants you to kill one of your fellow hostages whom he does not like with a knife he has given you. If you refuse his offer all of the hostages including the children and yourself will die. If you accept his offer then the others will die in the morning but you and the eight children will escape.

Submarine

You are the captain of a military submarine travelling underneath a large iceberg. An on-board explosion has caused you to lose most of your oxygen supply and has injured one of your crew who is quickly losing blood. The injured crew member is going to die from wounds no matter what happens.

The remaining oxygen is not sufficient for the entire crew to make it to the surface. The only way to save the crew members is to push the injured crew member out of an emergency hatch, killing him, but ensuring that there will be just enough oxygen for the rest of the crew to survive.

Vaccine Test

A viral epidemic has spread across the globe killing millions of people. You have developed two substances in your home laboratory. You know that one of them is a vaccine, but you don’t know which one. You also know that the other one is deadly

Once you figure out which substance is the vaccine you can use it to save millions of lives. You have with you two people who are under your care, and the only way to identify the vaccine is to inject each of these people with one of the two substances. One person will live, the other will die, and you will be able to start saving lives with your vaccine.

Yelling Person

You and a group of people are running away from a criminal gang. They are intent on killing all of you. You and some of the people have managed to out-run them and have sought refuge in an overgrown area of a park. At the entrance to the park, you hear the voices of the criminals who have come to find you.

One of the people hiding with you seems to be very nervous and they begin to yell loudly in panic. You grab them and press your hand over their mouth and face to block the sound. If you stop pressing, their yelling will summon the attention of the gang who will kill you and everyone hiding with you. To save yourself and the others you must smother the panicking person to death.

All dilemmas were followed by phrases specific to condition:

Judgment condition

Is it morally acceptable to [specific to the scenario]?

Would you do it?

Action condition

Are you going to [specific to scenario]?

If so, move the arm forward to [specific to scenario]. If not, then pull away [specific to scenario].

Act now.

APPENDIX B: Non-Moral Dilemmas

Maze

You are walking through a maze. You are approaching two diverging paths, each blocked by a round-shaped boulder.

By choosing the left path you will have to push a larger boulder to continue through the maze but you will get to the centre faster. By choosing the path on the right, you will get to push a lighter boulder but you will have further to travel until you can reach the centre of the maze.

Are you going to push the heavier boulder to the left in order to get to the centre of the maze faster?

If so, move the arm forward and push the heavier boulder. If not, then pull away from the boulder.

Taking the rubbish out

You intend to accomplish two things this afternoon: pushing the wheelie bin full of rubbish to the front of the garden ready for collection and watching some tv.

The weather is nice at the moment, but the forecast says that in a couple of hours it will start to rain. You very much dislike taking the bin out in the rain, but you don't care what the weather is like when you watch TV.

Are you going to push the wheelie bin to the front of the garden now before it begins to rain?

If so, move the arm forward and push the wheelie bin. If not, then pull away from the boulder.

Food prep

You are preparing pasta with fresh vegetables, and you are deciding on the order in which you will do the various things you need to do. You are in a big hurry.

At the moment you have a slight urge to cut vegetables. If you first start the water boiling and then cut the vegetables you will be done in twenty minutes. If you cut the vegetables and then start the water boiling you will be done in forty minutes.

Are you going to cut the vegetables first and then start the water boiling in order to satisfy your slight urge to cut vegetables?

If so, move the arm forward to start cutting the vegetables. If not, then pull away from them.

APPENDIX C:

Supplementary Study: Framing Effects

Method

Participants

Ninety-one participants (81 females, 12 males**,** M_{age} = 20.66, *SD* = 5.42 years, age range: 18 - 50) were recruited from the Plymouth University (School of Psychology) participant pool and participated for course credit. Participants completed the study online. This research received ethical approval from the Plymouth University Ethics Committee.

Procedure

Participants were randomly assigned to one of three conditions in which personal force dilemmas were presented to participants in the form of text-based vignettes. These were followed by; "*Is it morally acceptable to [specific to scenario]?"* (*N* = 28) or "*Would you do it?"* (*N* = 32) or *"Are you going to [specific to scenario]? Act now."* (*N* = 31). Participants were presented with a sample of eight of the 15 personal-force dilemmas incorporated into Experiment 1.

Results

Moral Responses

The proportion of utilitarian responses for personal-force dilemmas was higher for participants who were asked *"Are you going to [specific to scenario]? Act now."* (*M* = 0.40, *SD* = 0.24) than for those asked *"Would you do it?"* (*M* = 0.34, *SD* = 0.28) and those asked *"Is it morally acceptable to [specific to scenario]?"* (*M* = 0.33, *SD* = 0.31) (see Figure 1).

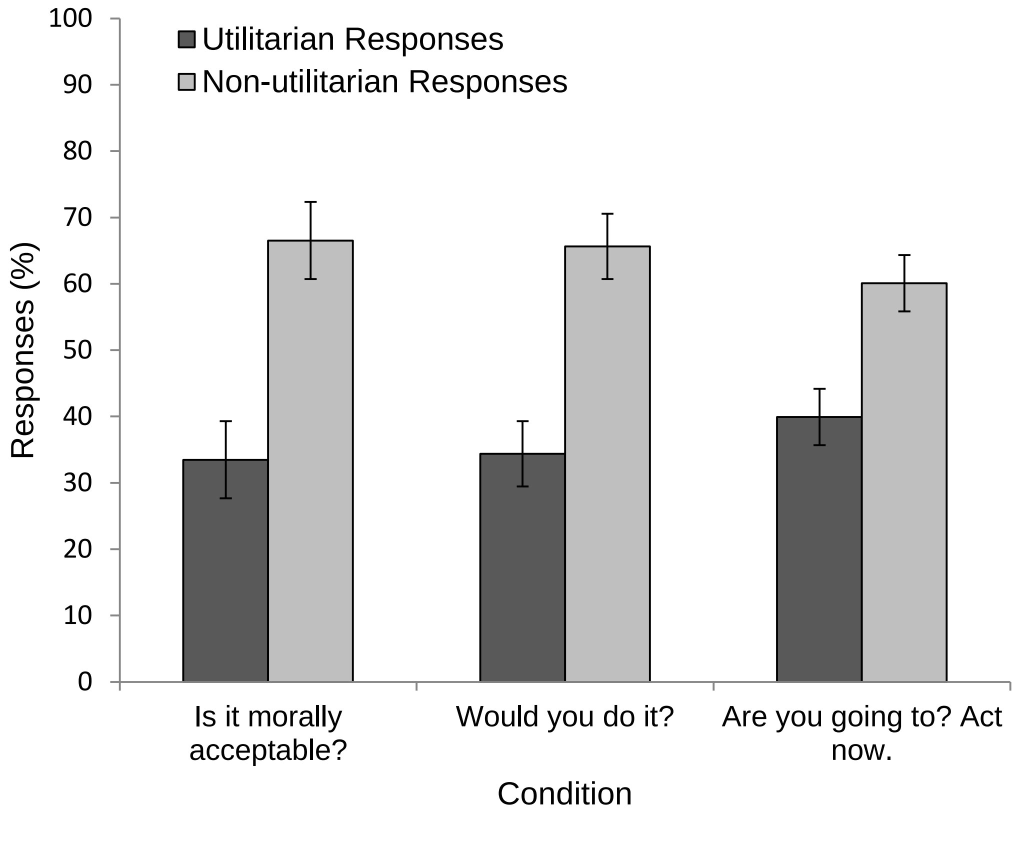

Figure 1. Responses (%) in the three framing conditions in response to eight of the original 15 personal-force dilemmas used in Experiment 1. Error bars represent +- 1 SE.

Although sample sizes in each condition were unequal, Levene's test indicated equal variances ($F = 1.90$, $p = .156$). A univariate ANOVA comparing the proportion of utilitarian responses in each of the framing conditions, revealed no main effect of condition, ($F(2,88) = 0.49$, $p = .615$).
